# A Connectome-Based, Corticothalamic Model of State- and Stimulation-Dependent Modulation of Rhythmic Neural Activity and Connectivity

**DOI:** 10.3389/fncom.2020.575143

**Published:** 2020-12-21

**Authors:** John D. Griffiths, Anthony Randal McIntosh, Jeremie Lefebvre

**Affiliations:** ^1^Krembil Centre for Neuroinformatics, Centre for Addiction and Mental Health, Toronto, ON, Canada; ^2^Department of Psychiatry, University of Toronto, Toronto, ON, Canada; ^3^Institute of Medical Sciences, University of Toronto, Toronto, ON, Canada; ^4^Rotman Research Institute, Baycrest Health Sciences, Toronto, ON, Canada; ^5^Department of Psychology, University of Toronto, Toronto, ON, Canada; ^6^Department of Biology, University of Ottawa, Ottawa, ON, Canada; ^7^Krembil Research Institute, University Health Network, Toronto, ON, Canada; ^8^Department of Mathematics, University of Toronto, Toronto, ON, Canada

**Keywords:** neural mass and field models, MEG magnetoencephalography, EEG electroencephalography, connectome, alpha rhythm, brain stimulation

## Abstract

Rhythmic activity in the brain fluctuates with behaviour and cognitive state, through a combination of coexisting and interacting frequencies. At large spatial scales such as those studied in human M/EEG, measured oscillatory dynamics are believed to arise primarily from a combination of cortical (intracolumnar) and corticothalamic rhythmogenic mechanisms. Whilst considerable progress has been made in characterizing these two types of neural circuit separately, relatively little work has been done that attempts to unify them into a single consistent picture. This is the aim of the present paper. We present and examine a whole-brain, connectome-based neural mass model with detailed long-range cortico-cortical connectivity and strong, recurrent corticothalamic circuitry. This system reproduces a variety of known features of human M/EEG recordings, including spectral peaks at canonical frequencies, and functional connectivity structure that is shaped by the underlying anatomical connectivity. Importantly, our model is able to capture state- (e.g., idling/active) dependent fluctuations in oscillatory activity and the coexistence of multiple oscillatory phenomena, as well as frequency-specific modulation of functional connectivity. We find that increasing the level of sensory drive to the thalamus triggers a suppression of the dominant low frequency rhythms generated by corticothalamic loops, and subsequent disinhibition of higher frequency endogenous rhythmic behaviour of intracolumnar microcircuits. These combine to yield simultaneous decreases in lower frequency and increases in higher frequency components of the M/EEG power spectrum during states of high sensory or cognitive drive. Building on this, we also explored the effect of pulsatile brain stimulation on ongoing oscillatory activity, and evaluated the impact of coexistent frequencies and state-dependent fluctuations on the response of cortical networks. Our results provide new insight into the role played by cortical and corticothalamic circuits in shaping intrinsic brain rhythms, and suggest new directions for brain stimulation therapies aimed at state-and frequency-specific control of oscillatory brain activity.

## 1. Introduction

A key characteristic of the fluctuations in extracranial electrical and magnetic fields measured by electroencephalography (EEG) and magnetoencephalography (MEG), resulting from the collective activity of large numbers of (primarily) cortical neurons, is that they are highly rhythmic. While the physiological origins and cognitive function of these rhythms remains unclear, their features are clearly highly labile: spatial location, frequency, and oscillatory power can vary considerably as a function of behaviour, cognitive processes, and disease. This suggests that not only the oscillations themselves, but also their fluctuations over time, space, and cognitive state play a key role in brain function. Moreover, multiple frequencies can coexist and interact, fluctuating in a highly correlated manner (Lisman and Jensen, 2013; Cohen, 2014). Understanding the mechanisms mediating the coexistence of these rhythms, as well as state-dependent changes in their properties, would yield important insight about how collective neural activity and synchronization phenomena, shaped by both sensory and recurrent inputs, mediate neural communication (Akam and Kullmann, 2012). “State” here simply refers loosely to gross cognitive/perceptual/neural activity regimes, as for example seen in the difference between low-frequency, high-amplitude oscillations observed at rest, and the relatively higher-frequency activity elicited by focused cognitive tasks. In the present paper we opt for the more neutral terms “idling” and “active” (as opposed to “rest” and “task”) to indicate these two dynamical regimes. To date only a few models in the literature have sought to explicitly capture transitions between these oscillatory states, and the dependence of certain neural processes on the current state (e.g., Cohen, 2014; Lefebvre et al., 2017). Experimental and theoretical results have shown that neural systems can undergo oscillatory transitions due to changes in stimuli statistics at various spatial scales (Jadi and Sejnowski, 2014; Hermes et al., 2015; Mierau et al., 2017), suggestive of a state-dependent flexibility in oscillatory coding and activity. Theoretical studies have highlighted multiple mechanisms that could mediate such fluctuations in the power spectrum (Brunel and Wang, 2003; Lefebvre et al., 2015), which is a principal focus of the present study.

Physiologically-based mathematical models of neural activity can be broadly divided into three types: *morphological models*—which describe passive and active ion fluxes within spatially extended neurons and circuits thereof; *single neuron models*—which describe spiking or firing rate activity of individual cells as point-processes with zero spatial extent; and *neural population models*—which describe the collective activity of large numbers of individual cells with low-dimensional equations. Neural population models are particularly suited to the study of noninvasive macroscopic signals such as MEG and EEG, for which the measurement physics require co-ordinated activity of thousands of individual cells to generate observable activity fluctuations. Some of the earliest neural population model formulations were due to Beurle (1956) and Freeman (1967), however the seminal formulations by Wilson and Cowan (1972), Nunez (1974), and Lopes da Silva et al. (1974) are those most in use still today, with important updates introduced by Jansen and Rit (1995), Jirsa and Haken (1996), Robinson et al. (1997, 2002), Liley et al. (2002), David and Friston (2003), and others. All of these approaches share the same basic structure, which can be summarized in terms of two principal mathematical operations (Freeman, 1975; Jirsa and Haken, 1997; Robinson et al., 2001): *pulse-to-wave conversion*—where pulsed incoming firing at the synapses of a neuronal population are converted to a continuous-valued post-synaptic membrane responses, and *wave-to-pulse conversion*—where average somatic post-synaptic depolarization is converted to outgoing firing rates.

The majority of neural population models that have been developed to account for the origins of large-scale brain rhythms can be grouped into two broad categories: (i) cortical-only and (ii) corticothalamic. A common feature of both of these is the assumption (as has been established by substantial physical and biophysical modelling and analysis) that the principal contributor to the strong extracranial signals measured by EEG and MEG are population-synchronous post-synaptic potentials in cortical layer V pyramidal cells. A second common feature of these, and indeed virtually all quantitative descriptions of oscillatory activity in neural systems, is the interplay between excitatory and inhibitory neural activity. The key difference between cortical-only and corticothalamic neural population models is where (i.e., which neural circuit, spanning which anatomical locations) the key excitatory-inhibitory interactions responsible for generating a given rhythmic pattern in observed M/EEG data is located. Cortical-only models propose to situate these circuit mechanisms directly within a cortical column (e.g., Jansen et al., 1993; Liley et al., 2002; David and Friston, 2003; Moran et al., 2011; Bastos et al., 2012), with formulations differing in precise details such as the number of interneuron populations and the presence of self-connections in inhibitory populations. Corticothalamic models (e.g., Robinson et al., 2001, 2002; Rowe et al., 2004; Cona et al., 2014; Saggar et al., 2015), in contrast, situate the relevant inhibitory circuit mechanisms in the thalamus rather than the cortex (specifically, the inhibitory GABA-ergic neurons of the thalamic reticular nucleus, which inhibit the relay nucleus), and in interactions between thalamic and cortical neural populations. These models thus attribute prominent spectral features such as low-frequency oscillations to delayed inhibition in long-range recurrent corticothalamic loops. For an excellent schematic summary of these different model classes and review of the theoretical landscape (see Liley, 2015).

Given the substantial bodies of empirical data from human and nonhuman physiological recordings supporting the existence of both the cortical-only and corticothalamic rhythmogenic mechanisms, it is highly likely that both play a role in the genesis of large-scale rhythmic activity observed in local field potentials and extracranial electromagnetic fields. Disambiguating the contribution of each to the different features of M/EEG signals, and how they might interact, is a challenging problem, however. Addressing this disconnect is one of the principal aims of the present study.

One of the major points of dispute between cortical-only and corticothalamic model types is the alpha rhythm. Alpha frequency (8–12 Hz) oscillations are a hallmark pattern of encephalographic activity (Berger, 1929; Adrian and Matthews, 1934). They have been linked to a wide variety of cognitive processes such as perception and attention, and their dynamic features (such as power and frequency) are also closely tied to changes in behaviour (Pfurtscheller and Da Silva, 1999; Mierau et al., 2017). Abnormal alpha activity is also involved in many neurological disorders such as depression, Parkinson's disease, and Alzheimer's disease (Uhlhaas and Singer, 2006; Rossini et al., 2007; Vanneste et al., 2018). Although still not uncontroversial, a broad range of experimental data point to the corticothalamic system as the most likely locus of the dominant alpha-frequency rhythmic activity seen in EEG and MEG (Lopes da Silva et al., 1974), as well as the phase relationship between alpha and other faster frequencies. In contrast, gamma frequency oscillations have been robustly tied to intracolumnar excitatory-inhibitory circuit mechanisms and active cortical information processing (Buzsáki and Wang, 2012; Womelsdorf et al., 2014). It remains an open question, however, how these two types of oscillatory activity (plus associated circuit mechanisms) shape large-scale neural dynamics, functional connectivity, and information integration in a state-dependent fashion.

A key experimental direction for investigating the dynamic properties and functional role of neural oscillations is to study the relationship between endogenous activity and responses to electromagnetic stimulation. This is not only critical for understanding the functional role of brain oscillations in general, but also for improving the efficacy of clinical applications of noninvasive brain stimulation, such as in the treatment of depression (Cocchi and Zalesky, 2018). Interestingly, a confluence of experiments with both intra-cranial and non-invasive stimulation have revealed frequency-specific responses, with low-frequency stimulation decreasing the excitability of stimulated tissue (Chen et al., 1997), and conversely higher frequency stimulation having the opposite effect (Dayan et al., 2013). Experiments in primates (Logothetis et al., 2010) and rodents (Liu et al., 2015) have indeed demonstrated that thalamic stimulation can be used to either activate or inactivate cortical networks in a frequency-dependent manner, opening new perspectives on the functional manipulation of cortical dynamics by exogenous signals.

To better understand state-dependent changes in oscillatory dynamics, their involvement in inter-area communication, and how they might be controlled by non-invasive stimulation, we present in this paper a novel connectome-based neural mass model that combines cortical and corticothalamic circuit mechanisms in a minimal and parsimonious fashion. As detailed in the *Methods*, the full model consists of a network of 68 interconnected nodes, representing brain regions derived from a commonly used parcellation covering most major cortical structures in the human brain. The dynamics of each node is described by an extension of the classic Wilson-Cowan (WC) equations (Wilson and Cowan, 1972), which we refer to as the “Cortico-Thalamic Wilson-Cowan” (CTWC) model. Our primary goal was to investigate how state-dependent inputs mediate changes in brain oscillations within multiple frequency bands, and how these spectral fluctuations shape functional connectivity. To do this, we began by examining the behaviour of a single isolated network node corresponding to an individual corticothalamic motif. We then moved on to examining collective dynamics, interactions, and the influence of stimulation within the whole-brain network.

## 2. Results

In the following sections, we first demonstrate that the CTWC model accurately reproduces several key characteristics of measured power spectra and functional connectivity from resting state MEG recordings. The model is then used to study the impact of sensory drive on brain rhythms, and how this serves to switch between low-frequency corticothalamically-driven vs. high-frequency cortically-driven oscillatory regimes. Finally, we show how the model predicts a number of empirical observations in humans and rodents on the relationship between brain state, periodic brain stimulation, and rhythmic entrainment of neural activity.

### 2.1. Alpha Rhythms Emerge From Delayed Recurrent Cortico-Thalamocortical Loops

In examining the dynamics of our corticothalamic model, we first considered the idling state, which we defined as being a state of minimal thalamic drive (see section 4) and thus reflecting dynamics in the steady state. Consistent with previous work (Lefebvre et al., 2017), this system produces a robust alpha rhythm with a spectral peak at approximately 10 Hz. In this idling regime, the higher frequency peaks in the power spectrum at beta and gamma frequencies reflect harmonics of the fundamental frequency (alpha), in line with previous reports using similar model architectures (e.g., Robinson et al., 2001).

**Figure 1:**
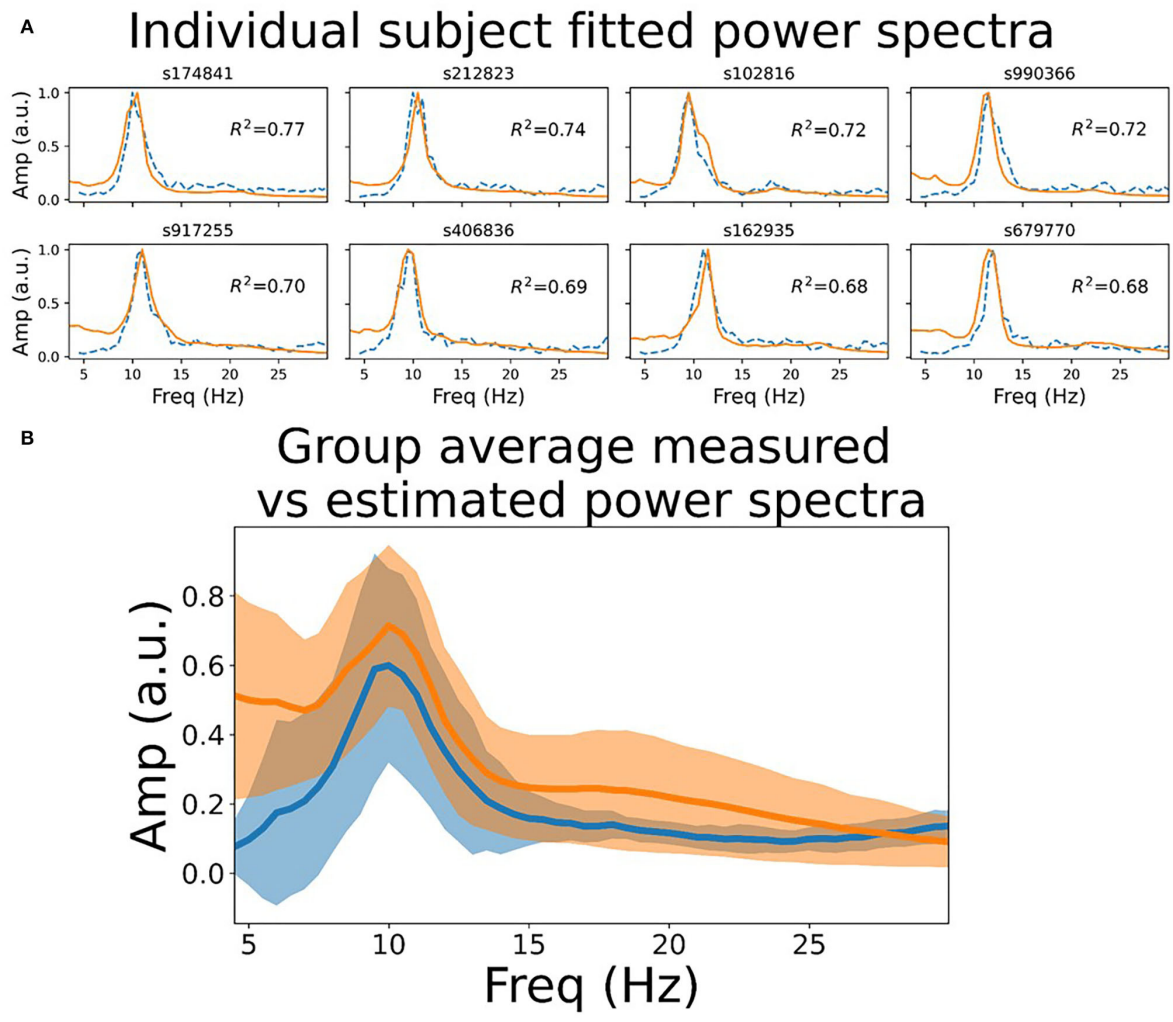
Resting state power spectrum fit to MEG data. **(A)** Sensor-averaged power spectrum from eight example HCP subjects' resting state MEG data (orange line), and corresponding simulated power spectrum from the CTWC model (dotted blue line). The simulated activity shows excellent fit to the empirical power spectrum (*R*^2^ between 0.6 and 0.8 in these examples), and accurately captures the alpha rhythm peak frequency in each subject. **(B)** Mean +/-1 standard deviation of the empirical and fitted power spectra for all 10 HCP subjects. Both the empirical and simulated power spectra also show 1/f scaling when plotted on log-log axes over a larger frequency range, as discussed in Supplementary Material, section 1.

To assess quantitatively the extent to which the model power spectra match empirical recordings in humans, we fit the model to MEG data in 10 subjects from the HCP WU-Minn consortium. Specifically, we computed the Pearson correlation between single-node model power spectra and channel-averaged empirical MEG power spectra, allowing two model parameters (*a*_*s*_ and *a*_*r*_; see section 4) to vary around their nominal values. As shown in Figure 1, this resulted in a good fit to the MEG data, with all subjects tested showing *R*^2^ ≥0.6. Interestingly, we see in empirical MEG data that there are larger differences in power spectra between subjects than between regions within a given subject (data not shown). This observation supports the modelling strategy of choosing a single set of parameters for each subject, and using those for all regions in the network; as opposed to using regionally varying parameter values. We return to the question of spatially varying spectral power below.

### 2.2. Phase Transition From Low-Frequency Idling to High-Frequency Active State

**Figure 2:**
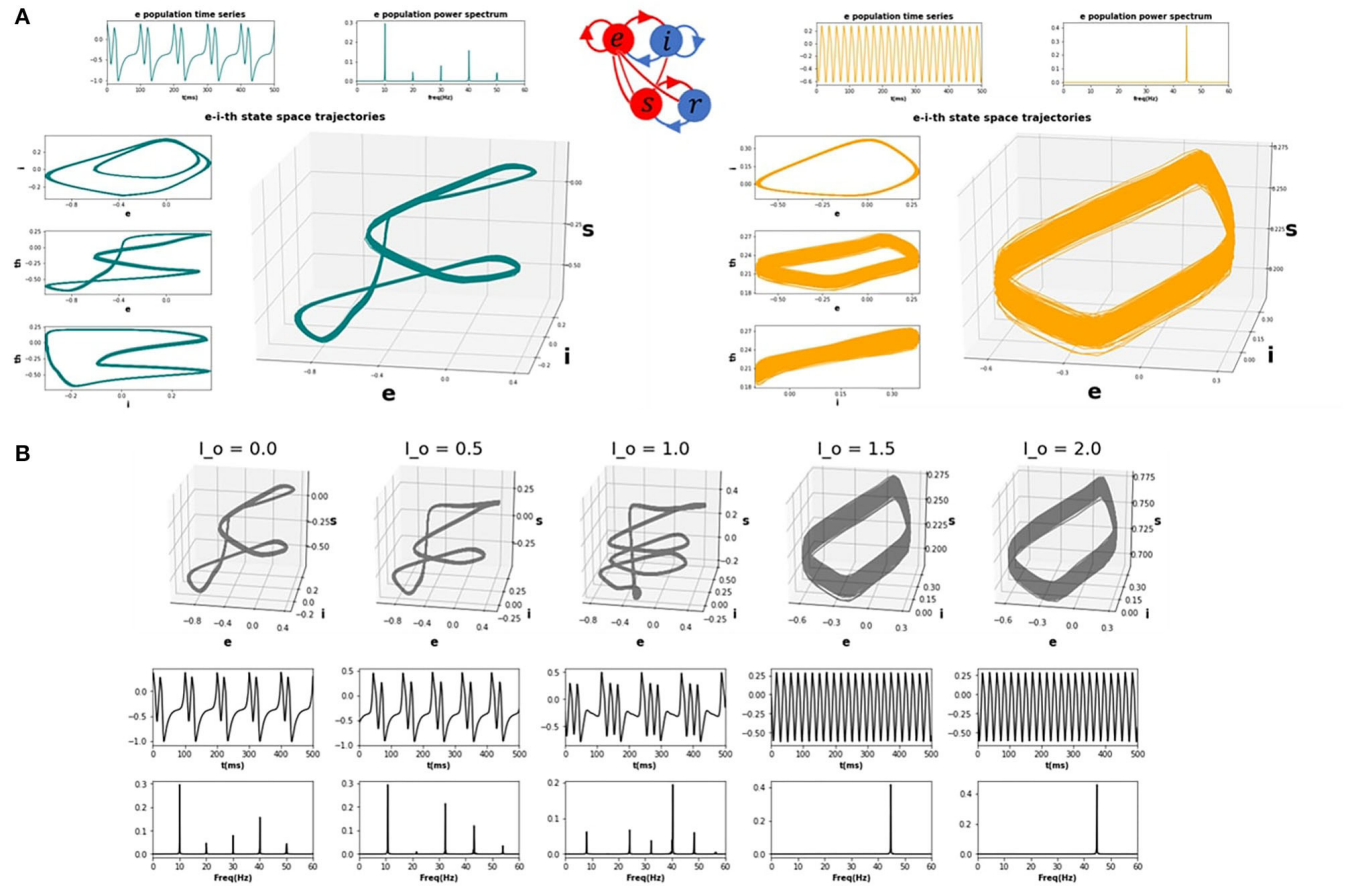
CTWC model phase space trajectories. **(A)** Exemplary phase space trajectories for a single corticothalamic unit in the idling (left; teal) and active (right; orange) regimes. Central 3D plot in each panel shows trajectories in the 3-dimensional phase space defined by the cortical excitatory (***e***), cortical inhibitory (***i***), and thalamic specific relay (***s***) population state variables. Orthogonal 2-dimensional views for each pair of state variables are shown on the left hand side. Panels above the trajectory figures show corresponding time series and power spectra for the ***e*** variable. The idling state regime (*I*_*o*_=0) is characterized by slow, nonlinear alpha-frequency (8–12 Hz) oscillations. Increasing the static sensory thalamic drive (here by setting *I*_*o*_=1.5) induces a phase transition into the active regime, where neural population activity is dominated by gamma-frequency (approximately 30 Hz) limit cycle dynamics. **(B)** Progression from idling to active regime. Sub panels show 3D phase plane trajectories, time series, and power spectra for incremental values of *I*_*o*_ between the idling and active states shown in **(A)**. As the system approaches the bifurcation point (*I*_*o*_ ≈ 1.4), the gamma attractor begins to manifest as a “twist” in the alpha limit cycle, which appears in the time series plot as embedded high-frequency ripples on the peak/trough of the oscillation. As *I*_*o*_ continues to be increase, eventually the low-frequency rhythm loses stability and the dynamics switches to a pure gamma oscillation.

Having characterized the dynamics within the idling state and the prevalence of alpha activity, we next asked how increasing the drive to the thalamic populations (either in one or multiple nodes) would impact the spectral properties of cortical activity. To emulate a task or “active” state, we thus increased the drive to the thalamic populations (see section 4) and observed the resulting behaviour. We first studied this systematically for a single isolated node. Figure 2 shows trajectories in the 3-dimensional phase space defined by the state variables *u*_*e*_, *u*_*i*_, and *u*_*s*_ (representing activity of excitatory cortical, inhibitory cortical, and thalamic specific relay nuclei, respectively), along with time series and power spectra for *u*_*e*_, which we take as a proxy for M/EEG source activity (Robinson et al., 2001; Moran et al., 2011). As the top left panel of Figure 2A shows, the system in the idling alpha-dominated regime (consistent with Figure 1) is characterized by a clean and highly stereotyped 10Hz limit cycle. Figure 2B and the right panels of Figure 2A then show how the system's dynamics and phase space are modified upon raising the static sensory input or drive parameter *I*_*o*_. We first observe (Figure 2B) with increasing *I*_*o*_ a gradual destabilization of the resting alpha rhythm, and a transfer of oscillatory power from alpha to higher frequencies. This destabilization is characterized in the three-dimensional phase space by an increase in the number and regularity of short, rapid excursions (“twists”) within the alpha limit cycle, which in the time series plots appear as nested higher-frequency “ripples” within the 10 Hz base oscillation. Eventually, after a bifurcation point around *I*_*o*_=1.3 is crossed, the system shifts completely to a noisier, low(er) amplitude gamma-frequency limit cycle, with a clear peak in the power spectrum observed at 30 Hz. In line with a confluence of empirical studies (Jadi and Sejnowski, 2014), this high-frequency component of the power spectrum reflects the fast-paced interplay between excitatory and inhibitory neural populations, and is generated locally within the cortical compartments of each network node. Due to the nature of the corticothalamic circuit motif we considered here, this increased thalamic drive also represents an increased engagement of cortical excitatory and inhibitory populations, that are now recruited for active processing of afferent inputs.

### 2.3. Influence of Regionally Focal Thalamic Drive

**Figure 3:**
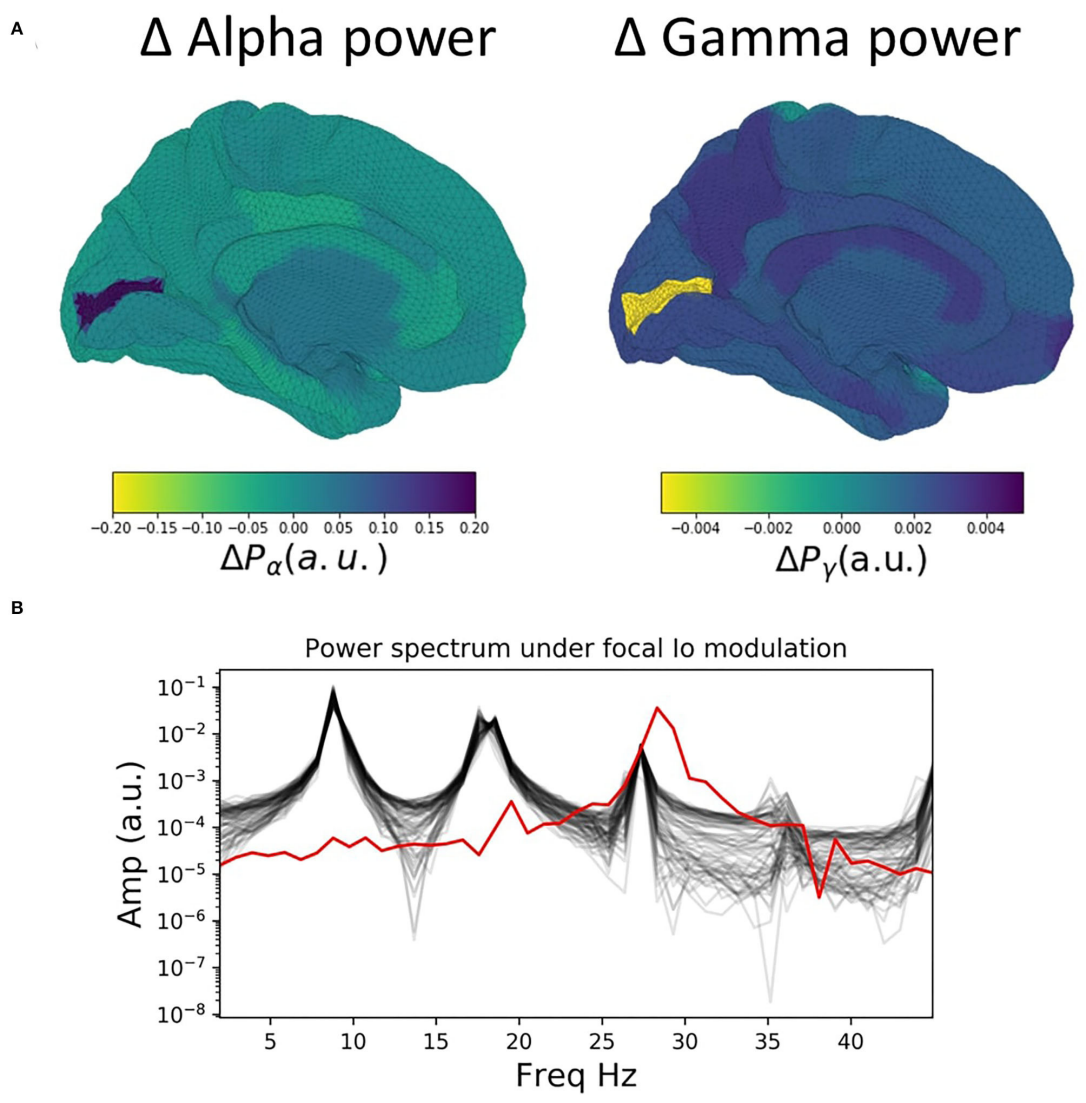
Influence of focal thalamic drive in a whole brain network. **(A)** Surface renderings of the regional change (Δ) in alpha and gamma power from baseline to active state for all brain regions. Increased sensory thalamic drive in visual cortex results in suppression of alpha and enhancement of gamma band activity, reminiscent of the patterns routinely observed in M/EEG studies of visual-evoked gamma. **(B)** Power spectra for baseline values of the tonic thalamic relay nucleus driving term (*I*_*o*_=0), and for focal increase (*I*_*o*_=1.5) in left visual cortex (lV1). Red lines show power spectra for the lV1 node; black lines for the other 67 nodes. Note the prominent increase in relative gamma power and decrease in relative alpha power in lV1 when that node's *I*_*o*_ value is increased.

We now extend the observations and insights obtained from the single-node case considered in the previous section to the case of whole-brain network behaviour. Figure 3 shows time series, power spectra, and brain-wide plots of the change (Δ) in alpha and gamma power for simulations where *I*_*o*_ is modulated focally for a single node (left V1) in the 68-node network. The suppression of alpha power and enhancement of gamma power with increasing drive is clearly evident in the surface plots and lower power spectrum (Figure 3A).

### 2.4. Functional Connectivity

Given the salient differences in oscillatory dynamics observed in the idling and active states, we investigated how these different oscillatory regimes shaped inter-area interactions in a whole-brain network context. To do this, we compared functional connectivity, as measured by amplitude-envelope correlations (AECs) of band-limited power time series (Hunt et al., 2016), in model-generated time series and empirically measured MEG data.

**Figure 4:**
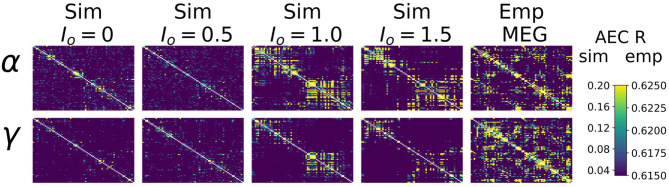
AEC FC vs. *I*_*o*_. Upper panel: Alpha- and Gamma-frequency AEC matrices for 4 values of *I*_*o*_ (*I*_*o*_=0./0.5/1.0/1.5), alongside the empirically-measured MEG gamma-frequency AEC matrix.

**Figure 5:**
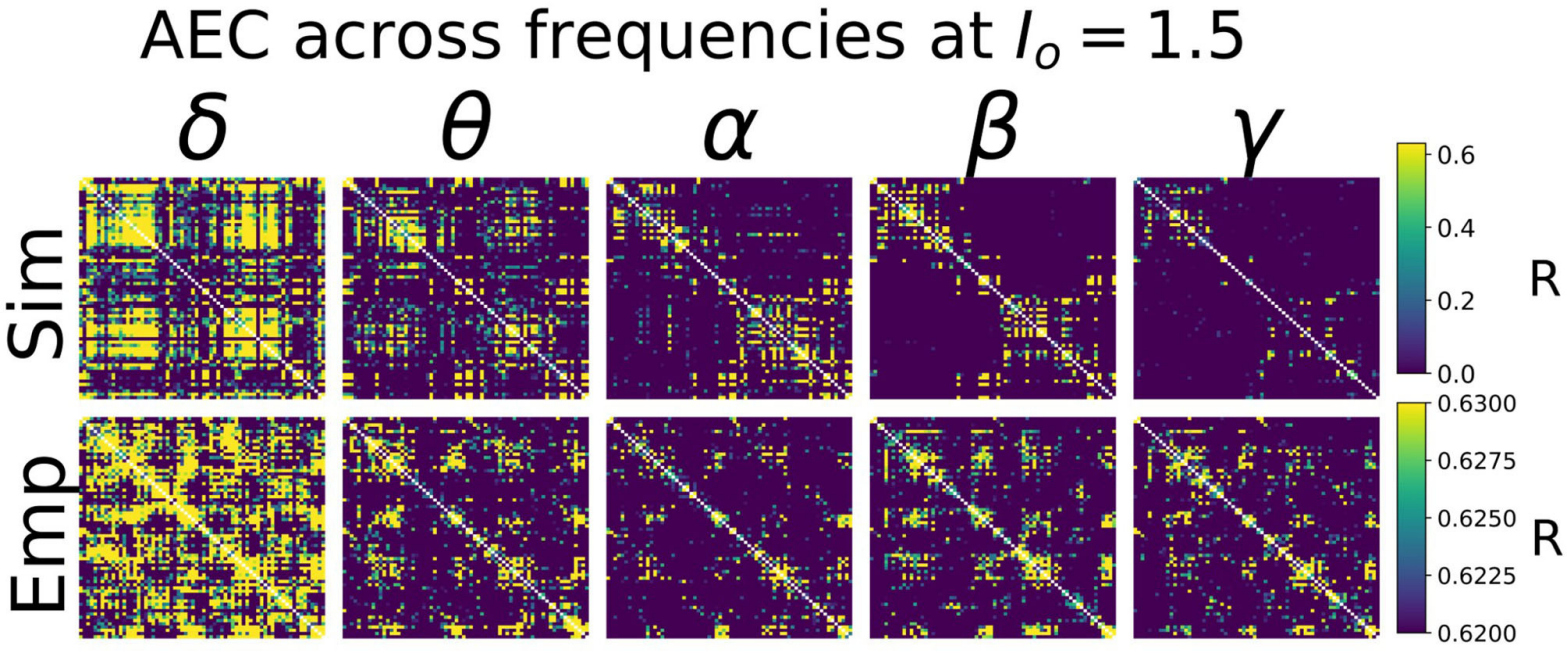
AEC FC vs. Frequency. Shown are AEC FC matrices at five different canonical frequency bands—δ (0.5–4 Hz), θ (4–8 Hz), α (8–12 Hz), β (13–30 Hz), and γ (30–45 Hz)—from simulations (top row) and from empirical MEG data (bottom row). In both simulated and empirical data, lower frequencies (δ and θ) show less spatial specificity and more tendency toward random connectivity patterns. Note that the more compressed AEC range in empirical than simulated AEC data is due to the application of orthogonal leakage correction (Colclough et al., 2015) in analyses of MEG data. Global parameters *I*_*o*_ and *g* were 1.5 and 5, respectively—see Supplementary Material, section 2 for further exploration of these values.

Heuristically, moving from an isolated node to a network of coupled nodes results in two important changes in the “environment” experienced by each node. First, the overall or time-averaged activity level of a given brain region will be higher when there are inputs from other regions than when there are no inputs. Second, depending on the behaviour of the incoming signals from other regions, that node may experience periodic or otherwise temporally structured driving inputs. This, in turn, may lead to the emergence of synchronization and collective behaviour throughout the system due to processes of entrainment or resonance, possibly also accompanied by bifurcations. As shown in Figure 4, we found idling and active states in the model to be characterized by quite different functional connectivity profiles. The idling state exhibits relatively weaker and spatially non-specific AEC patterns at both alpha and gamma frequencies. In contrast, as the increased static drive *I*_*o*_ pushes the system into the gamma-dominated active state, both alpha- and gamma-frequency AEC matrices increasingly come to display the kind of spatial structure characteristic of empirically measured AEC (as well as by various other M/EEG, fMRI functional connectivity, and indeed anatomical connectivity metrics). Specifically, the active state shows a stronger tendency for spatially nearby regions to show high correlations (as indexed in the AEC matrices by the “halo” of high connectivity values around the leading diagonal), and the classic two-block hemispheric structure with stronger intra- than inter-hemispheric correlations. Interestingly, although the two characteristic frequency regimes within the model are in the alpha- and gamma- ranges, it also captures some properties of AEC outside of these ranges. Figure 5 shows empirical vs. simulated AEC for the full range of classic M/EEG frequencies: delta (0.5–4 Hz), theta (4–8 Hz), alpha (8–12 Hz), beta (13–30 Hz), and gamma (30–60 Hz). As can be seen, moving from low to high frequencies within the active regime is also accompanied by sparser and more spatially structured correlation patterns.

Our findings described thus far have shown that active and idling states are characterized by different spectral signatures, and that functional connectivity is differentially expressed in a frequency-specific way in these two states. Next, we examined the effects of periodic stimulation on ongoing cortical activity. That is, we asked: can the temporal structure neural activity be tuned by exogenous signals in a frequency-specific way?

### 2.5. Susceptibility to Entrainment by Exogenous Stimulation Is State-Dependent

Having characterized idling and active states, their dominant spectral features and how they impact functional connectivity, we investigated how *exogenous* periodic stimulation shapes the power spectrum of the system and engages ongoing oscillations. Numerous studies over the last few decades have used stimulation paradigms of various kinds to access circuit function and interfere with neural communication (Thut et al., 2011; Helfrich et al., 2014; Cecere et al., 2015). One of the most robust findings is that entrainment of ongoing brain oscillations is state-dependent, and that susceptibility to control is tuned by ongoing brain fluctuations - an effect that has also been reproduced with modelling (Neuling et al., 2013; Alagapan et al., 2016) and shown to involve stochastic resonance (Herrmann et al., 2016). Given the ability of our model to switch between different states and express multiple frequencies, we subjected cortical populations to exogenous periodic stimulation and monitored the spectral response. Specifically, we again studied an isolated cortico-thalamo-cortical motif (i.e., a single network node), and computed the peak power and frequency as a function of stimulation intensity and frequency. Through this process, we identified resonances and entrainment regimes (so-called *Arnold Tongues*) and thus measured the susceptibility of our model to entrainment. While oftentimes confused with one another, *resonance* refers to the enhancement of power when the stimulation frequency is in the vicinity of the system's natural frequency, while *entrainment*, refers to the phase locking of the system's response to the driving signal (Herrmann et al., 2016).

**Figure 6:**
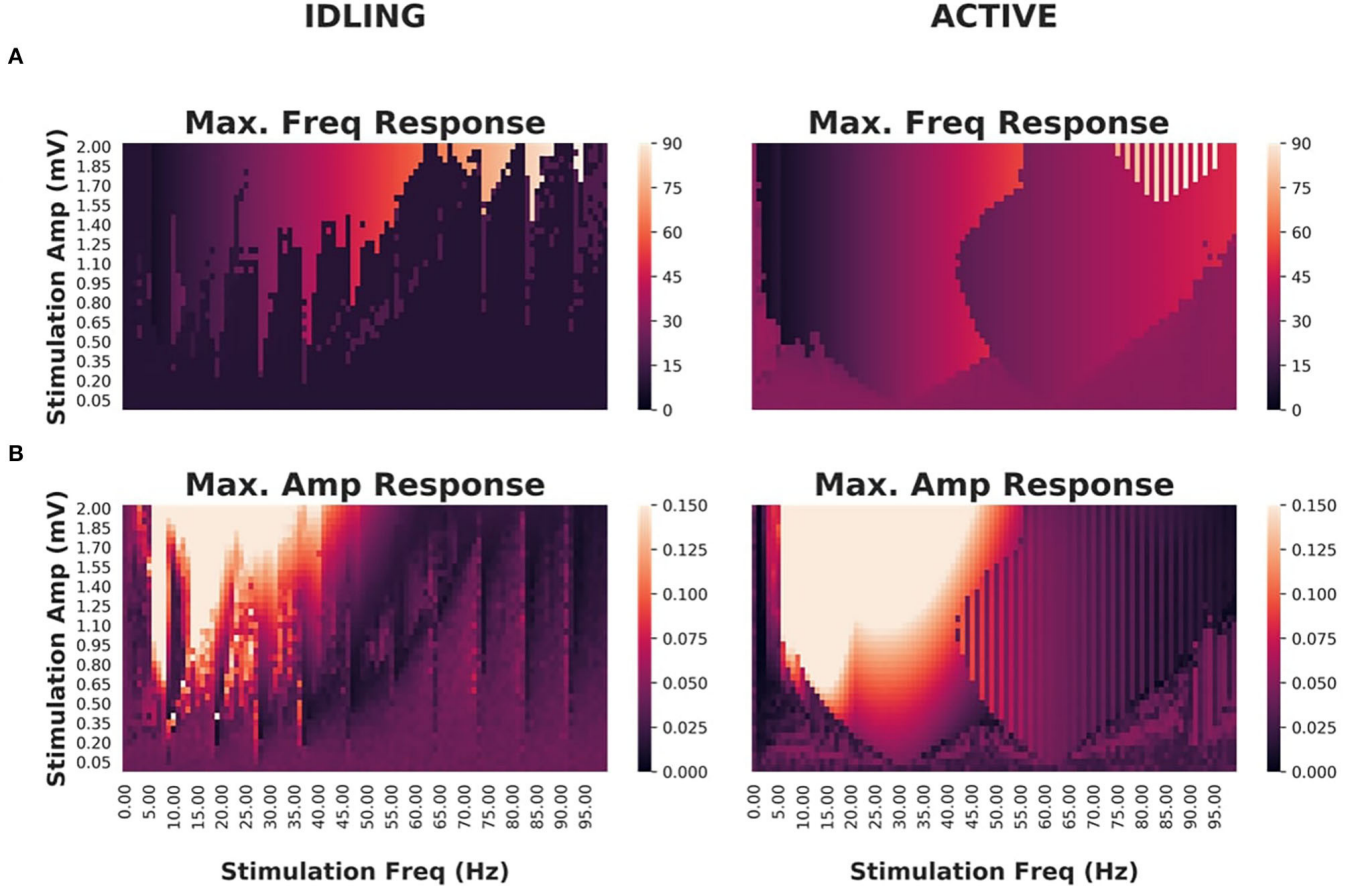
Effects of periodic brain stimulation on corticothalamic loop dynamics **(A)** Maximum frequencies displayed by the cortical excitatory population of an isolated cortico-thalamocortical loop (CTWC model, single node) in response to periodic (sine wave) stimulation of varying amplitudes (y axes) and frequencies (x axes). In the idling regime, an Arnold Tongue structure is clearly seen centered on the natural frequency (approximately 10 Hz): As the stimulation frequency moves away from the natural frequency, greater stimulation amplitude is required to achieve entrainment at the stimulation frequency. In the active regime, a broader and shallower Arnold Tongue structure is again seen, centered on the natural frequency (this time approximately 30 Hz). Compared to the idling state, entrainment at the stimulation frequency is easier to achieve (requires lower amplitude stimulus) in the active than the idling regime. **(B)** Maximum amplitudes displayed by cortical excitatory populations. Here again the amplitude response patterns match quite closely the Arnold Tongues seen in the maximum frequency responses.

As shown in Figure 6, idling and active states exhibited significant differences in their responses to stimulation and susceptibility to entrainment. Narrower Arnold Tongues were observed in the idling state compared to the active state, indicating that the suppression of alpha power in the active state facilitates phase locking of intrinsic dynamics with the stimulation signal. Specifically, only high intensity stimulation would provoke a shift in the peak frequency in the idling state. In the active state, the prominent gamma oscillations were easily suppressed and replaced by the frequency of the driving stimulus. This is in line with converging evidence indicating that intrinsic attractors limit the effect of perturbations, while irregular or high frequency content is more malleable (Lefebvre et al., 2017).

## 3. Discussion

The aim of the present study was to investigate the mechanisms underlying state-dependent changes in oscillatory activity at the whole-brain scale, as well as the influence of fluctuations in spectral activity on functional connectivity. We have presented a novel connectome-based neural mass model that combines the two primary rhythmogenic mechanisms typically studied in large-scale brain network modelling: intracolumnar microcircuits and corticothalamic loops. This is an extension of previous work, that studied the behaviour of the basic corticothalamic motif in isolation (Griffiths and Lefebvre, 2019). Here we have embedded this corticothalamic unit into a whole-brain network, with anatomical connectivity derived from diffusion MRI tractography. Our model reproduces a variety of known features of human M/EEG recordings, including spectral peaks at canonical frequencies and functional connectivity structure that is shaped by the underlying anatomical connectivity. Using this model, we have studied how thalamic drive mediates a shift in oscillatory regime, provoking a transition between alpha and gamma dominance in the power spectrum, and found that these oscillations have a differential impact on functional connectivity patterns. We found that spatially structured inter-area functional connectivity (as measured by band-limited power amplitude envelope correlations), particularly at higher frequencies (gamma, beta, and alpha to a lesser extent), are a hallmark of the active state. To better understand how these state- and frequency-specific dynamics are impacted by exogenous stimulation, we applied cortical periodic stimulation of various amplitudes and frequencies, eliciting endogenous resonances both across the corticothalamic loop and within cortex. Our analysis confirms that, as compared to the idling state, the active state is more susceptible to entrainment by exogenous signals, as it shows wider and shallower Arnold Tongues. In contrast, the idling state's deep and narrow Arnold Tongues indicate that the system has a strong preference for its natural frequency when in this regime, and will respond only to exogenous signals close to that frequency or its harmonics.

### 3.1. Relation to Previous Work

The work presented here builds on previous work of several authors in a number of ways. Most directly, the isolated CTWC neural mass model (without the whole-brain white matter connectivity introduced here) was recently introduced in Griffiths and Lefebvre (2019). Previous to that we have also studied resonance behaviour, response to stimulation, and state-dependence in corticothalamic circuits and generic feedback oscillators (Alagapan et al., 2016; Hutt et al., 2018a; Park et al., 2018). We emphasize however that the core mathematical and conceptual component of the CTWC model presented in the present paper and in our earlier work—namely the generation of slow M/EEG rhythms through a delayed inhibitory cortico-thalamo-cortical recurrent circuit, has been used extensively by multiple groups for several decades. One of the largest and most comprehensive bodies of work on this is due to P. Robinson and colleagues, beginning with the introduction in Robinson et al. (1997) of a PDE wave equation reformulation of the integro-differential cortical neural field model of Wright and Liley (1996), drawing on earlier work of Lopes da Silva et al. (1974), Jirsa and Haken (1996), and others. This model was then augmented with thalamic reticular and relay nuclei and their recurrent connections with the cortex (Robinson et al., 2001), and the resultant corticothalamic neural field model has been studied extensively over the past two decades—both analytically and numerically, and in partial differential, ordinary differential, and linearized equation forms, as well as being extended into the domains of epilepsy, Parkinson's, sleep and arousal, plasticity, and brain stimulation (e.g., Robinson et al., 2001; Rowe et al., 2004; Breakspear et al., 2006; Van Albada et al., 2010; Roberts and Robinson, 2012; Fung and Robinson, 2013; Abeysuriya et al., 2015; Abeysuriya and Robinson, 2016; Müller et al., 2017; Mukta et al., 2019). Our approach in the present paper differs from this family of models in two key ways. First, rather than the second-order equations of motion for the time-evolution of membrane voltage used by Robinson and many others (Lopes da Silva et al., 1974; Jansen et al., 1993; David and Friston, 2003), we began with the classic Wilson-Cowan equations (Wilson and Cowan, 1972) to describe local interactions between excitatory and inhibitory neural population activity levels in a cortical region. In taking this route we are building on the extensive body of work using Wilson-Cowan equations as a model for cortically-generated gamma frequency oscillations. Additionally, a notable advantage of our choice to use Wilson-Cowan equations, rather than for example the neural mass version of the Robinson neural field wave equations, is that they are mathematically and computationally simpler. For example, whereas the minimal system of coupled first-order ODEs for a single corticothalamic motif with the Robinson model would have eight state variables (because the original equations are second-order), the CTWC model has only four state variables per corticothalamic unit. Whilst second-order dynamics may be important in neural mass models for capturing specific aspects of damped evoked response waveforms, it is unclear whether they are necessary for describing oscillatory activity. The second key difference in our work from other research to date using the Robinson model is that rather than using an integro- or partial-differential equation formulation of a continuum neural field to represent spatio-temporal propagation of activity across the cortex (Jirsa and Haken, 1996; Robinson et al., 1997, 2016; O'Connor and Robinson, 2004; Nunez and Srinivasen, 2006; Gabay and Robinson, 2017), here we chose to follow the connectome-based neural mass modelling methodology (Honey et al., 2007; Ghosh et al., 2008; Deco et al., 2009; Ritter et al., 2013; Sanz Leon et al., 2013; Sanz-Leon et al., 2015; Cabral et al., 2014; Spiegler et al., 2016) of defining a discrete network of point-process neural masses, interconnected via long-range white matter fibers whose density was estimated from non-invasive diffusion MRI tractography. This combination of the cortico-thalamocortical circuit with the large-scale anatomical connectivity bears some similarity to the work of some other authors (e.g., Freyer et al., 2011; Cona et al., 2014; Saggar et al., 2015; Bensaid et al., 2019), but the present study is the first to apply this directly to the key questions of state-dependence, alpha suppression, functional connectivity, stimulation, and their relation to empirical M/EEG data. Notably, this network-based approach allowed us to harmonize the analysis of functional connectivity in simulated and empirical MEG data. In this we followed the approach of Abeysuriya et al. (2018) and Hadida et al. (2018) in our use of the bandpass-filtered amplitude envelope correlations (Brookes et al., 2011; Hunt et al., 2016), and that line of work is perhaps the closest of recent modelling studies to the present one. Abeysuriya et al. (2018) studied the role of inhibitory synaptic plasticity in a connectome-based network of Wilson-Cowan equations. As in the present study, these authors evaluated their model in terms of its ability to accurately reproduce empirically measured MEG AEC matrices (although they restricted their focus to only to alpha-frequency AECs). The relatively simpler (as compared with our new CTWC) model used by these authors consisted of a cortical Wilson-Cowan ensemble, tuned to have a natural frequency in the alpha range. This stands somewhat in contrast to our new model, which features a *gamma* frequency-tuned Wilson-Cowan ensemble, combined with an *alpha* frequency-tuned cortico-thalamocortical motif. This additional two-component structure allows our model to exhibit more complex behaviours, such as alpha-mediated inhibition and state-switching, as well as a rich repertoire of potential oscillation and frequency-specific synchronization patterns. The question of whether and to what extent human M/EEG alpha activity is generated by corticothalamic (as in e.g., the present study and much of the above-cited work by Robinson and colleagues), or within intracortical microcircuits (as in e.g., Liley et al., 2002; David and Friston, 2003; Moran et al., 2011; Abeysuriya et al., 2018) remains a live and important one however. Recent years has also seen growing interest in a third potential type of system-level (low-frequency) rhythmogenic mechanism which can be broadly described as *network eigenmodes* (Robinson et al., 1997, 2016; Nunez and Srinivasen, 2006; Cabral et al., 2014; Tewarie et al., 2019). The proper evaluation and assessment of these hypotheses around cortical rhythmogenesis shall most likely require a close interaction between novel empirical work and hypothesis-generating computational models to properly settle. It is also important to bear in mind here that there is no a priori reason (apart from explanatory parsimony) to suppose a single mechanism for generation of rhythms (Nunez and Srinivasen, 2006). Indeed, it may be functionally advantageous for the brain to generate the same frequency through a variety of mechanisms. If this were determined to be the case, then interaction across different frequency-generating mechanisms would be a key question for future work.

### 3.2. The Alpha Rhythm as a Suppression Mechanism

The transition from idling to active state in our model is initiated by the gradual increase of a tonic sensory drive term, *I*_*o*_, that effectively hyperpolarizes the thalamic relay nucleus, and thereby destroys the slow 10 Hz alpha rhythm generated by the cortico-thalamocortical loop. Once the alpha oscillation is removed in this way, the gamma rhythm generated by intracortical excitatory-inhibitory interactions comes to the fore. One interpretation of this phenomenon is that alpha resonance, mediated by corticothalamic loops, plays an inhibitory role - through which slow oscillatory corticothalamic activity suppresses and dominates higher frequency cortical activity. This alpha-as-suppression-mechanism theory speaks to a major question in the field of M/EEG cognitive neuroscience: what is the functional role of alpha? Specifically, the enhancement of alpha activity during disengagement of the cortical network (such as during quiescence, sleep, anaesthesia, and withdrawal of sensory stimulation) suggests that alpha oscillations implement a functionally inhibitory signal, and represent a top-down shift toward internal encoding through suppressing the activity of task-irrelevant areas (Klimesch et al., 2007). In contrast, faster frequencies, such as those found in the beta and gamma range, are found in states of arousal and sensory recruitment, suggesting a positive, excitatory role of faster neural oscillatory states. In our model, the less spatially-resolved structure of functional connectivity in the alpha vs. the gamma range—at all *I*_*o*_ values, but particularly for *I*_*o*_ ≥1.4—does support this perspective. From this point of view, a key feature of our model is its characterization of the relationship between corticothalamically-generated and cortically-generated rhythms. In particular, the corticothalamic alpha dominates in the idling state, and can be understood as suppressing the intrinsic rhythmic activity in the cortical ensemble, which can be “released” with sufficient sensory (or perhaps neuromodulatory) drive. This simple circuit mechanism therefore captures a widely used theoretical concept in M/EEG cognitive neuroscience concerning the functional role of alpha activity. On this account, alpha acts as a mechanism for selectively gating and attentionally biasing sensory inputs. This phenomenon is also observed in EEG studies on the effects of anaesthesia, where low frequency activity becomes increasingly dominant with higher doses of propofol (Supp et al., 2011). This effect is observed concurrently with apparent attenuation of sensory inputs, for example in reduced amplitude and increased latency of somatosensory evoked potentials (SEPs). Recent work in mouse models has also shown that driving thalamic circuits with alpha-frequency activity causes widespread depression of cortical activity; whereas stimulating at higher frequencies (e.g., gamma) causes widespread increase in both baseline activity and the spatial spread of the stimulation influence (Liu et al., 2015).

Interestingly, in our analyses we observed that the active-state model AEC patterns actually showed closer resemblance to empirical resting-state MEG AEC patterns than the idling-state AEC patterns. This is somewhat unexpected because resting-state MEG power spectrum was unequivocally better fit by a CTWC model in the idling, alpha-dominated regime. This result suggests that in the brain, during the rest or idling state, alpha power is strong and AEC functional connectivity is largely random. In contrast, in the active state, alpha power is relatively weaker, and AECs are more local and segregated. Functional connectivity is thus facilitated in the high-drive state, when the alpha-generating loop is inhibited, and dynamics are driven by cortico-cortical E-I interactions. In the state of low-drive, the alpha rhythm is highly prominent and neural activity is largely asynchronous (i.e., low functional connectivity). In the state of high drive, the alpha rhythm has been suppressed, and functional connectivity is high. Together, these observations suggest that the alpha rhythm plays a suppressing role in large-scale brain dynamics. We hypothesize that this may be a general feature of alpha activity, with regional communication facilitated by being in the active state, and resting activity characterized by a constant interplay and balance between the idling state and the active state. The presence of regular intermittent switching between idling and active states could also account for the fact that the perspective outlined above is partly in contradiction with the empirical data shown in Figures 4, 5—specifically that resting state MEG data do show somewhat spatially structured connectivity patterns. In this case, intermittent switching over the period of a 5–10 min resting state MEG recording would result in some spatially structured correlations due to a mixing of the two regimes. This picture is broadly consistent with related observations from Schirner et al. (2018), who developed a “hybrid” neural mass modelling approach aimed at integrating concurrently recorded resting state fMRI and EEG data. These authors found, consistent with the “gating by inhibition” hypothesis (Jensen and Mazaheri, 2010), that long-range input in whole-brain simulations was decreased during states of high alpha power, and increased again when alpha power decreased. Their brain network model simulations provide a mechanistic explanation of gating by inhibition, by demonstrating how increased alpha power leads to increased feedback inhibition of excitatory populations. The modulation of population firing resulting from this mechanism was proposed to explain empirically observed alpha phase- and power-dependent firing rate modulations, as well as the well-known anticorrelation between alpha power and fMRI BOLD signal.

### 3.3. Conclusions and Future Directions

To conclude: we have developed a novel whole-brain connectome-based neural mass model that incorporates corticothalamic and intracortical rhythmogenic mechanisms. This model reproduces qualitatively multiple features of MEG-measured neural activity. Importantly, our model also lends some insight into the way that corticothalamically-generated alpha rhythms could play a functional role in the organization of brain dynamics, by suppressing high-frequency cortical activity associated with cognitive engagement and information processing. Future work shall investigate further questions of subcortical parcellation and integration, model fitting, and compare alternative rhythmogenic mechanisms directly against each other. Importantly, future work should also investigate the significance of intersubject variability in anatomical connectivity on network dynamics. Although we demonstrated here our model's ability to fit individual subjects' power spectra through small variations in thalamic kinetic parameters, it was beyond the scope of the present study to incorporate individualized anatomical connectivities. One of the exciting and promising aspects of connectome-based neural mass modelling is the possibility of constructing individualized computational models using a subjects' own diffusion MRI tractography. However at this point in time the extent to which this does actually deliver improvement in computational model accuracy remains an open question for the field (for recent work relevant to this, see Abeysuriya et al., 2018; Zimmermann et al., 2018). Finally, we emphasize that neither our specific CTWC model, nor the broader alpha-as-suppression-mechanism concept, constitute a universal account of all alpha-frequency rhythms seen in the M/EEG or other recording modalities. Indeed we consider the most likely scenario to be that multiple, dissociable mechanisms contribute independently a proportion of the information and measured signal in that part of the frequency spectrum (Nunez and Srinivasen, 2006). Here we have, building on previous work, made we believe some progress in characterizing the dynamic properties of one of these candidate mechanisms.

## 4. Methods

Our modelling approach follows the now-standard whole-brain connectome-based neural mass modelling paradigm (Honey et al., 2007; Ghosh et al., 2008; Deco and Jirsa, 2012; Sanz-Leon et al., 2015), where dynamic units are placed at node locations as defined by a gray matter parcellation, and coupled with an adjacency matrix (anatomical connectome) defining the presence and associated strengths of long-range white matter fibers interconnecting region pairs. The anatomical connectome used in the present study, derived from group-average tractography streamline counts, was constructed from analyses of the human connectome project (HCP) WU-Minn consortium diffusion-weighted MRI (DWI) corpus (Glasser et al., 2013; Sotiropoulos et al., 2013). For details of this, see the below section 4.2. In the model, activity at each node is driven by background noise and/or exogeneous stimulation. Complete mathematical formulation and implementation details are given in the section 4.1. Simulated nodal time series from the model can be understood as approximations of regionally averaged source-space MEG signals. To assess the performance of the model in reproducing key features of empirically measured human brain dynamics, we additionally conducted new analyses of the HCP WU-Minn resting-state MEG corpus (Larson-Prior et al., 2013). These are described in section 4.3.

### 4.1. Corticothalamic Model

**Figure 7:**
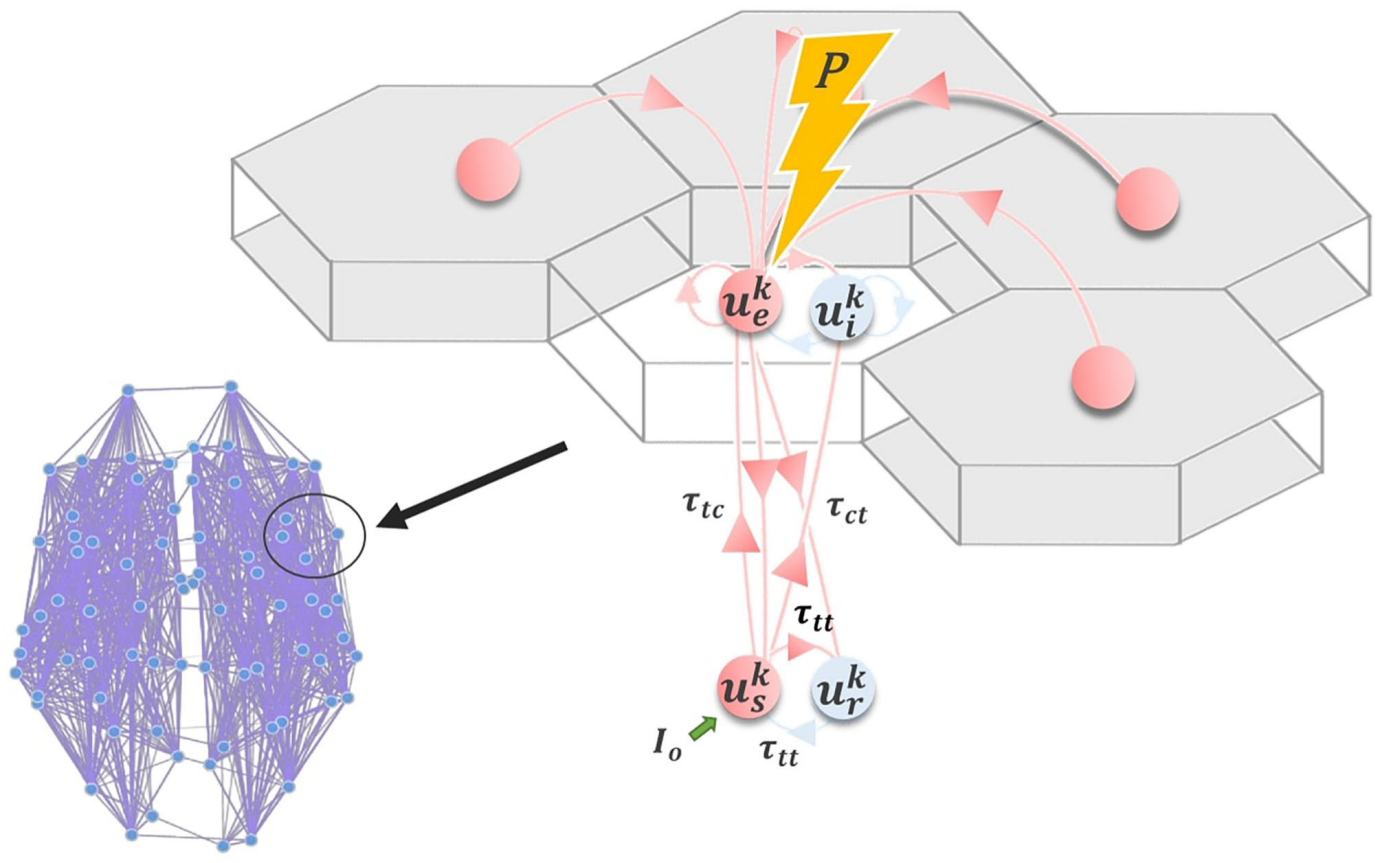
Corticothalamic model. Schematic of the corticothalamic model structure. Cortical (*u*_*e*_, *u*_*i*_) and thalamic (*u*_*s*_, *u*_*r*_) populations interact through a delayed feedback loop. Entrainment of the network activity through electromagnetic stimulation P applied to *u*_*e*_ depends on the amplitude and frequency of the stimulation pulse, as well as the network state, controlled by *I*_*o*_.

Following other authors (Robinson et al., 2001; Breakspear et al., 2006; Freyer et al., 2011), we employ a model for neuronal dynamics at each node that incorporates both cortical and thalamic neural populations. The model describes a four-component cortico-thalamo-cortical motif, consisting of excitatory (**u**_*e*_) and inhibitory (**u**_*i*_) cortical neuronal populations, coupled to thalamic reticular (**u**_*r*_) and specific relay (**u**_*s*_) nuclei (Figure 7). Both relay and reticular nuclei receive inputs from the cortical excitatory population, following a corticothalamic conduction delay τ_*ct*_. However only the relay nucleus sends excitatory input back to the cortex; again received following a delay τ_*ct*_=τ_*tc*_. The reticular nucleus, which is widely known to have an inhibitory influence on other thalamic regions (Zhang and Jones, 2004), plays a similar role to the cortical inhibitory population, inhibiting the relay nucleus and thereby generating oscillatory dynamics. Note that thalamic relay nuclei in this and similar corticothalamic models may be either First- or Higher-Order (Sherman, 2006), depending on the part of cortex in question. Our model does not distinguish between First- and Higher-Order relay nuclei, or recapitulate the details of their specific circuitry. It simply assumes that every cortical region has reciprocal excitatory projections from one or other thalamic relay nucleus, which in turn receives inhibitory projections from the thalamic reticular nucleus.

As defined, our node-level model consists of a Wilson-Cowan oscillatory neural population, embedded in a delayed inhibitory feedback loop mediated by corticothalamic and thalamocortical connections. The full network-level model thus consists of a set of *N* such local units of this kind, coupled using the connectivity matrix **W** (anatomical connectome). The system of stochastic delay-differential equations governing the time-evolution of neural activity within the network can be summarized as follows:

(1) $$\begin{array}{l} \begin{array}{cc} D_{p}u_{p}^{j} & =\;\underset{\begin{array}{c} \text{neural}\\ \text{interactions} \end{array}}{\underset{︸}{G\left[u_{p}^{j}\right]}}\;+\underset{\begin{array}{c} \text{static and time-}\\ \text{varying stimulation} \end{array}}{\underset{︸}{{\text{S}}_{p}P^{j}+{\text{S}}_{i}I_{o}^{j}}}\;+\underset{\begin{array}{c} \text{background}\\ \text{noise} \end{array}}{\underset{︸}{\sqrt{2D}\xi_{p}^{j}}}\; \end{array} \end{array}$$

where the temporal differential operator $D_{p}=\left(1+\alpha_{p}^{-1}\frac{d}{dt}\right)$ incorporates population time constants α_*p*_, and $u_{p}^{j}$ refers to the mean somatic membrane activity of the neural population *p* ∈ {*e, i, r, s*} within one cortico-thalamic module *j* across the brain-scale network of *N*=68 nodes. Irregular and independent fluctuations are also present in the network, modeled by the zero-mean Gaussian white noise processes $\xi_{p}^{j}$ with standard deviation *D*. The neural interaction term $G\left[u_{p}^{j}\right]$ in Equation (1) can be further broken down into

(2) $$\begin{array}{l} G\left[{\text{u}}^{j}\left(t\right)\right]=\;\underset{\begin{array}{c} \text{intra-}\\ \text{cortical} \end{array}}{\underset{︸}{\text{A}F\left[{\text{u}}^{j}\left(t\right)\right]}}+\underset{\begin{array}{c} \text{cortico-}\\ \text{thalamic} \end{array}}{\underset{︸}{\text{B}F\left[{\text{u}}^{j}\left(t-\tau_{ct}\right)\right]}}\;+\underset{\begin{array}{c} \text{intra-}\\ \text{thalamic} \end{array}}{\underset{︸}{\text{C}F\left[{\text{u}}^{j}\left(t-\tau_{tt}\right)\right]}}\\ \;+\underset{\begin{array}{c} \text{cortico-}\\ \text{cortical} \end{array}}{\underset{︸}{\text{K}Q}} \end{array}$$

where the matrices

(3) $$\begin{array}{l} \begin{array}{c} \text{A}=\left(\begin{array}{cccc} \;g_{ee} & g_{ei} & 0 & 0\\ g_{ei} & g_{ie} & 0 & 0\\ 0 & 0 & 0 & 0\\ 0 & 0 & 0 & 0\; \end{array}\right),\text{B}=\left(\begin{array}{cccc} \;0 & 0 & 0 & g_{es}\\ 0 & 0 & 0 & g_{is}\\ g_{re} & 0 & 0 & 0\\ g_{se} & 0 & 0 & 0\; \end{array}\right),\text{C}=\left(\begin{array}{cccc} \;0 & 0 & 0 & 0\\ 0 & 0 & 0 & 0\\ 0 & 0 & 0 & g_{rs}\\ 0 & 0 & g_{sr} & 0\; \end{array}\right) \end{array} \end{array}$$

respectively specify the gains (connection strengths) of intracortical, corticothalamic, and intrathalamic interactions within a node. Intrathalamic and corticothalamic/thalamocortical connections are retarded by conduction delays τ_*ct*_=20ms and τ_*tt*_=5ms, respectively. The matrix

(4) $$\begin{array}{l} \begin{array}{c} \text{K}=\left(\begin{array}{cccc} \;g_{cc} & 0 & 0 & 0\\ 0 & 0 & 0 & 0\\ 0 & 0 & 0 & 0\\ 0 & 0 & 0 & 0\; \end{array}\right) \end{array} \end{array}$$

specifies the global gain applied to all afferent activity *Q* arriving from other cortical neural populations. The four rows and columns of **A**, **B**, **C**, and **K** correspond to the four neuronal populations *p* ∈ {*e, i, r, s*}, respectively, in the cortico-thalamic unit motif. In the present model we assume for simplicity that afferent activity only impacts on the cortical excitatory population *u*_*e*_; and so only the upper left entry in **K** is nonzero. The afferent activity in *Q* is a time-delayed summation of *u*_*e*_ at all other nodes in the network

(5) $$\begin{array}{l} \begin{array}{c} Q^{j}\left(t\right)=\overset{N}{\underset{k=1}{\sum }}{\text{W}}^{jk}F\left[u_{e}^{k}\left(t-{\text{T}}^{jk}\right)\right] \end{array} \end{array}$$

where **W** and **T** are cortical white matter connectivity and conduction delay matrices, both of which are derived from empirical diffusion-MRI tractography reconstructions (see below). For the latter, the cortico-cortical conduction delay matrix **T** = **L**/*cv* is calculated from a matrix of measured (average) fiber tract lengths **L**, assuming a fixed conduction velocity *cv*=4 m/s. The sigmoidal response function *F* in Equations (2) and (5) specifies the nonlinear response of a neural population to incoming inputs as follows

(6) $$\begin{array}{l} h\begin{array}{c} F\left[u\right]={\left(1+exp\left(-\beta \left(u-\sigma \right)\right)\right)}^{-1} \end{array} \end{array}$$

The matrices

(7) $$\begin{array}{l} \begin{array}{c} {\text{S}}_{p}=\left(\begin{array}{cccc} \;1 & 0 & 0 & 0\\ 0 & 0 & 0 & 0\\ 0 & 0 & 0 & 0\\ 0 & 0 & 0 & 0\; \end{array}\right),{\text{S}}_{i}=\left(\begin{array}{cccc} \;0 & 0 & 0 & 0\\ 0 & 0 & 0 & 0\\ 0 & 0 & 0 & 0\\ 0 & 0 & 0 & 1\; \end{array}\right) \end{array} \end{array}$$

in Equation (1) parameterize the impact on the four sub-populations *e, i, r, s* within a node of the time-varying exogenous input *P* (representing periodic brain stimulation such as rTMS or TACS) and static input *I*_*o*_ (representing here state-dependent sensory drive). Again, in the present study we only consider exogeneous inputs to impact the cortical excitatory populations, and so only the upper left entry in **S**_**p**_ is nonzero. Similarly, *I*_*o*_ is for present purposes only considered to impact the thalamic relay nucleus, and so only the lower right entry of *S*_*i*_ is nonzero. The exogeneous periodic signal *P* here is given by the simple sinusoidal function

(8) $$\begin{array}{l} \begin{array}{c} P^{j}=M^{j}\sin\left(2\pi \omega t\right) \end{array} \end{array}$$

with frequency ω and intensity *M*. The constant state-dependent drive $I_{o}^{j}$ to thalamic relay populations serves as a control parameter indexing idling vs active states (see below). This static input current can be thought of as a tonic level of sensory (e.g., visual) drive, although it could also reflect a static influence of ascending (e.g., noradrenergic) neuromodulatory drive, reflecting the level of engagement in a perceptual or cognitive task. Irrespective of its cause, the idling or rest-like state is defined as the dynamics resulting from setting $I_{o}^{j}$=0; i.e., in the absence of this constant thalamic input. The active state, in contrast, is defined by a greater engagement of thalamic nodes, and hence $I_{o}^{j}\geq$0 for active nodes. In both of these cases, nodes within the network may be differentially recruited by a given task, thus being activated while others remain inactivated. This represents an intermediate point between the extreme cases where all nodes are either active or inactive.

With the described structure, and right choice of parameters, our system generates alpha (8–12 Hz) oscillations due to the presence of delayed inhibition, as well as gamma (30–120 Hz) oscillations resulting from the cortical activity and interactions, and also in a limited domain of parameter space shows coexistence of both of these features. As has been demonstrated previously (Griffiths and Lefebvre, 2019), increasing the thalamic drive parameter past a critical point triggers suppression of resting state alpha oscillations, and results in a greater susceptibility of cortical neural populations to entrainment by exogenous inputs or noninvasive stimulation. In addition, this transition to the active state is accompanied by an increase in high-frequency (i.e., gamma) activity. As such, the thalamic drive can be seen as a control parameter, controlling the power of alpha and gamma oscillations, as well as tuning the response to exogenous inputs.

Nominal parameter values and definitions from the above-specified system of equations are summarized in Table 1. Parameter values were chosen based on a combination of parameter sets found in the literature (Jadi and Sejnowski, 2014) as well as in our previous work (Lefebvre et al., 2017; Hutt et al., 2018b; Griffiths and Lefebvre, 2019), where oscillatory activity is spontaneously and simultaneously observed in both the gamma and alpha band.

**Table 1:** Model parameters.

**Name**	**Unit**	**Nominal value**	**Description**
*a*_*e*_	*ms*	0.3	Cortical excitatory population time constant
*a*_*i*_	*ms*	0.5	Cortical inhibitory population time constant
*a*_*s*_	*ms*	0.2	Thalamic relay nucleus time constant
*a*_*r*_	*ms*	0.2	Thalamic reticular nucleus time constant
*i*_*e*_	*mV*	−0.35	Cortical excitatory population constant input
*i*_*i*_	*mV*	−0.3	Cortical inhibitory population constant input
*i*_*s*_	*mV*	0.5	Thalamic relay nucleus constant input
*i*_*r*_	*mV*	−0.8	Thalamic reticular nucleus constant input
τ_(*ct*/*tc*)_	*ms*	20	Corticothalamic / Thalamocortical conduction delay
τ_*tt*_	*ms*	5	Thalamo-thalamic conduction delay
*I*_*o*_	*mV*	0.	Static sensory/neuromodulatory drive
*dt*	*ms*	0.1	Integration step size
*w*_*ee*_		0.5	Excitatory-excitatory gain
*w*_*ei*_		1	Excitatory-inhibitory gain
*w*_*ie*_		−2	Inhibitory-excitatory gain
*w*_*ii*_		−0.5	Inhibitory-inhibitory gain
*w*_*er*_		0.6	Excitatory-reticular gain
*w*_*es*_		0.6	Excitatory-relay gain
*w*_*si*_		0.2	Relay-inhibitory gain
*w*_*se*_		1.65	Relay-excitatory gain
*w*_*rs*_		−2	Reticular-relay gain
*w*_*sr*_		2	Relay-reticular gain
*D*_(*e, i, r, s*)_		0.0001	Noise standard deviation for all populations
*g*		5	Global connectivity scaling factor
β		20	Activation function gain parameter
σ		0	Activation function threshold parameter

The system was numerically integrated using a stochastic Euler-Maruyama scheme, implemented in Python. Simulations were carried out on an 8-core Ubuntu 14.04 machine. Run time scaled approximately linearly: each 4-s simulation ran in approximately 4 s real time. The single-node simulations in Figure 1 were run for 4 s simulated time each. The whole-brain simulations in Figures 4, 5 were run for 5 min each, corresponding to the duration of the resting-state MEG data recordings. Subsequent parameter space explorations in Figure 6, Supplementary Figures 3, 4 were run for 20 s each. Supplementary Material, section 3 examines the dependence of simulation features on simulation length, and serves to justify the use of 20 s for the PSE sweeps (which reduces by an order of magnitude the computation time as compared to the full 5 min runs). All code and processed data used in this study is freely available at https://github.com/GriffithsLab/ctwc-model, along with additional notes and comments. A version of the model has also been developed for direct use within The Virtual Brain modelling and neuroinformatics platform (TVB; www.thevirtualbrain.org, Ritter et al., 2013; Sanz Leon et al., 2013; Woodman et al., 2014). Our model produces regional time series for each network node, as specified by the anatomical parcellation. These represent the collective activity of neural populations within that region, and as such correspond to signals estimated from MEG source reconstruction. Subsequent power spectrum and functional connectivity analyses of simulated activity time series therefore proceeded identically to that for MEG data, and are described in section 4.3.

### 4.2. DWI Data Analyses

The anatomical connectivity matrices used in this paper were constructed using diffusion- and T1-weighted MRI data from the HCP WU-Minn consortium (Glasser et al., 2013; Sotiropoulos et al., 2013; Van Essen et al., 2013). For detailed descriptions of the MR acquisition parameters and processing pipeline (see Glasser et al., 2013; Sotiropoulos et al., 2013). The HCP WU-Minn corpus consists of multimodal imaging and behavioural data from 1,200 healthy, young (ages 20–40) subjects. The tractography analysis described below was applied to a 700-subject subset of the full sample; and the connectivity matrix used for simulations in the present paper was calculated from an average over these 700 subjects. The HCP WU-Minn minimal diffusion pipeline (Glasser et al., 2013) consists of gradient nonlinearity correction, eddy current correction, boundary-based registration, and reorientation of diffusion data to the T1 image, and gradient vector rotation. The outputs of this preprocessing pipeline were the starting point for our diffusion data analyses. Using the minimally preprocessed diffusion data, we performed whole brain deterministic tractography reconstructions using the *Dipy* software library (Garyfallidis et al., 2014), following a methodology modeled closely on that of Hagmann et al. (2008) and Cammoun et al. (2012). ODFs were computed at each white matter voxel using a DSI tissue model. Streamlines were initiated from 60 regularly-spaced grid points within each voxel on the gray-white matter interface (as determined from coregistered freesurfer surfaces), and propagated using the EuDX algorithm (Garyfallidis, 2012). Streamlines not terminating at the gray-white matter interface, or having lengths >250 mm or <10 mm, were discarded. Subjects' streamline sets were segmented using the Lausanne scale-1 parcellation (Hagmann et al., 2008; Daducci et al., 2012), computed individually for every subject from their freesurfer reconstructions using algorithms from the connectome mapping toolkit (Daducci et al., 2012). All surface-based parcellations were then converted to image volumes and resliced to diffusion space for streamline segmentations. For each parcellation, the interconnecting streamlines for every ROI combination were determined using a logical AND operation. Each segmented streamline set was counted and its average length computed, resulting in streamline count and length matrices for each subject. The simulations described in the present paper were computed using group-average tract length matrices (divided by conduction velocity to convert to conduction delay), and group-average streamline count matrices, with the latter first being log-transformed to adjust for the DWI tractography over-estimation bias (Abeysuriya et al., 2018).

### 4.3. MEG Data Analyses

MEG analyses were performed using 10 randomly selected subjects from the HCP WU-Minn corpus (Larson-Prior et al., 2013), using the MNE software library (Gramfort et al., 2013, 2014). The specific analyses done were based on a modified version of the analysis pipeline developed by Engemann and colleagues (https://github.com/mne-tools/mne-hcp), which implements a full source space analysis, beginning with the HCP preprocessed sensor-space data. Key outcome variables from this pipeline for the present study were whole-brain functional connectivity matrices and spectral power maps, derived from regional source time series estimates. We opted to implement a complete analysis here rather than use the high-level pipeline outputs provided with the HCP WU-Minn corpus, as we needed complete control over the process. In particular, we needed to (a) use the same parcellation in the MEG as in the tractography analyses, and (b) ensure identical analyses were done on empirical and simulated MEG regional time series. Regarding the first of these: as in the tractography analyses, the parcellation used for MEG analyses was the Lausanne2008 scale 1—but with subcortical nodes (brainstem, basal ganglia, thalamus) excluded. Note this is in fact identical to the freesurfer *aparc* parcellation (but reordered and renamed).

Source time series were extracted for all vertices within a parcel using an L2 minimum-norm inverse solution and averaged, yielding one representative time series per parcel. To maximize robustness of these signals, this was operation was repeated five times, with 30-second windows each (Larson-Prior et al., 2013). Subsequent analysis of these regional time series proceeded identically (except for leakage artifact corrections; see below) for both the empirical and simulated MEG data. We first computed power spectra for each region using Welch's method (as implemented in the *scipy.signal* library; 256-sample windows, with 128-sample overlap). We then studied functional connectivity within the system using the band-limited power amplitude envelope correlation (AEC) method (de Pasquale et al., 2012; Hunt et al., 2016). For this, regional time series from each of the 5 windows were bandpass-filtered into six canonical frequency bands: delta (0.5–4 Hz), theta (4–8 Hz), alpha (8–12 Hz), beta (12–30 Hz), and low gamma (30–45 Hz) (Hunt et al., 2016). For the empirical MEG data, the symmetric orthogonalization method (Colclough et al., 2015) was applied to the bandpass-filtered time series to remove potentially spurious correlations due to source leakage; this step was omitted for simulated time series as they are by construction free of leakage artifacts. Amplitude envelopes were then computed as the real part of the analytic signal obtained from the Hilbert transform of the band-limited time series. Pearson correlations between these regional bandpass-filtered amplitude envelope time series were computed, and averaged over the 5 windows. Finally, the resultant region-to-region AEC matrices at each frequency band were averaged over subjects. Because our simulations used a normative (rather than subject-specific) anatomical connectivity, these analyses were conducted only once on the simulated MEG data, and this was compared to the group-averaged MEG data using Pearson correlations to evaluate the performance of the model.

## Data Availability Statement

Complete code for simulations and data analyses described in this article is available at: https://github.com/griffithslab/ctwc-model.

## Ethics Statement

The studies involving human participants were reviewed and approved by University Health Network. Written informed consent for participation was not required for this study in accordance with the national legislation and the institutional requirements.

## Author Contributions

JG and JL conceived and designed study. JG coded and ran numerical simulations and data analyses. JG, JL, and AM wrote the paper. All authors contributed to the article and approved the submitted version.

## Conflict of Interest

The authors declare that the research was conducted in the absence of any commercial or financial relationships that could be construed as a potential conflict of interest.
