# Supplementary material for: A Connectome-Based, Corticothalamic Model of State- and Stimulation-Dependent Modulation of Rhythmic Neural Activity and Connectivity
Source: Front Comput Neurosci. 2020 Dec 21;14:575143. doi: 10.3389/fncom.2020.575143 (PMC7779529; doi:10.3389/fncom.2020.575143)
Supplement: Supplementary file 1 [file Data_Sheet_1.pdf]

Supplementary Material for "A connectome-based, corticothalamic model of state- and stimulation-dependent modulation of rhythmic neural activity and connectivity"

1 POWER LAW SCALING IN SIMULATED SPECTRA

Figure 1 in the main text shows example power spectra from the CTWC model that were fitted to individual-subject resting state MEG data. In that figure the data is shown on linear frequency (x) and (normalized) amplitude (y) scales, because the focus is on low-frequency power spectrum features (particularly alpha-frequency spectral peaks at at 8-12Hz). The simulated data also shows "1/f" scaling in the power spectrum, as we demonstrate in this section. Figure S1 below shows the same data from Figure 1, plotted on log-log scales. In figure S2, we then re-plot the group average data from Figure S1B along with 1/f trend lines for two different power law exponent values. The trend lines are define by the equation $10^b * 1/F^x$, where x is the exponent and b is an intercept or linear offset term.

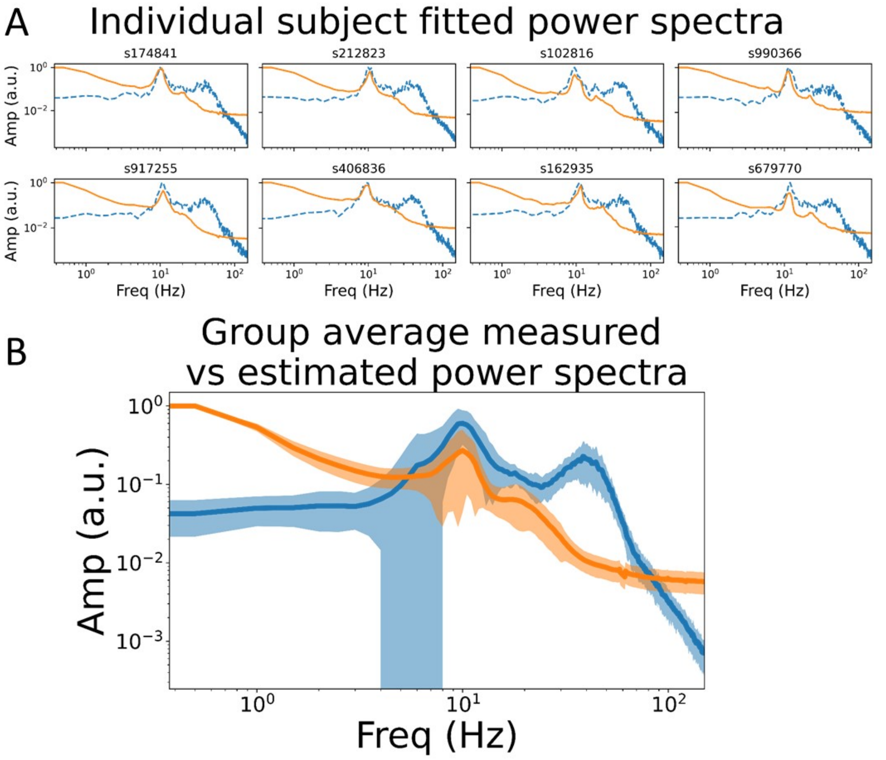

Figure S1. ***Empirical vs. Simulated Power Spectra, log-log-scale***. This figure shows exactly the same data presented in Figure 1 of the main text, but on a log-log axis and with a correspondingly larger frequency range. A) Eight example single subject, sensor-averaged empirical MEG (orange) and optimally matching simulation (blue) power spectra. B) Group average empirical and best-fit simulated power spectra.

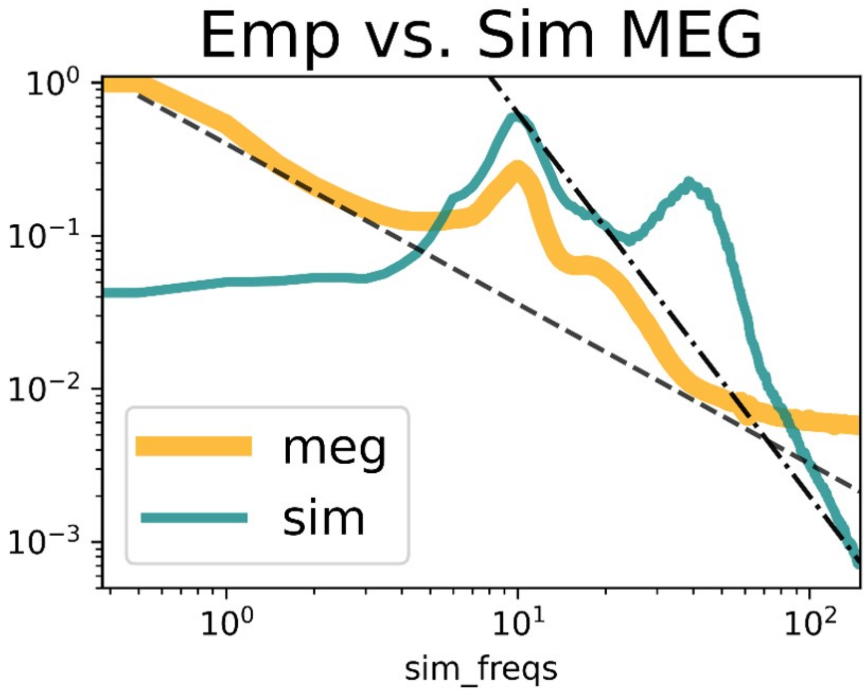

Figure S2. ***Group averaged Empirical vs. Simulated Power Spectra, log-log-scale, with exponent lines.*** These are the same group-averaged curves from Figure S1b, with the shaded distributions removed and exponent lines added. The empirical MEG data shows a power law exponent of roughly 1.05, as indicated by the dashed line. The simulated data does show power law scaling, with an exponent of 2.5, but only beginning at roughly 10Hz, as indicated by the steeper dash-dot line.

The empirical MEG data shows a clear 1/f trend with an exponent of approximately 1.05. The simulated data, in contrast, is fairly flat in the low-frequency part of the spectrum, and starts to show 1/f trend with an exponent of 2.5 above around 10Hz. The two spectral peaks in the simulated data represent the two oscillatory regimes (alpha-dominated and gamma-dominated) demonstrated in the main text Figure 2.

2 PARAMETER-SPACE EXPLORATION AND SIMULATED DATA FITS

The simulations presented in this paper all used a fixed global coupling parameter of $g = 5.0$, along with a variety of I_o values from 0 to 1.5. In this section we present the justification for the values of g and I_o selected. We conducted a parameter space exploration (PSE) over all possible combinations of I_o and g, within ranges of 0-20 and 0-20, respectively. The results of these PSEs are shown in Figures S3 and S4. Figure S3a and S4 show the fit between empirical and simulated data. This was computed as the Pearson correlation (R) between the vectorized upper triangles of the (symmetric) amplitude envelope correlation matrices from empirical and simulated MEG time series - repeated over all five frequency bands (delta, theta, alpha, beta, gamma; see main text Figure 5), and averaged. Note that we applied the orthogonal leakage correction to the empirical but not the simulated AEC matrices, since the simulated data have zero source leakage by construction. These simulated-empirical fit (R) values are shown for each parameter combination as heatmap intensity values in Figure S3a, and in Figure S4 as y axis height in line plots for a subset of the columns (Figure S4a) and rows (Figure S4b) of Figure S3a.

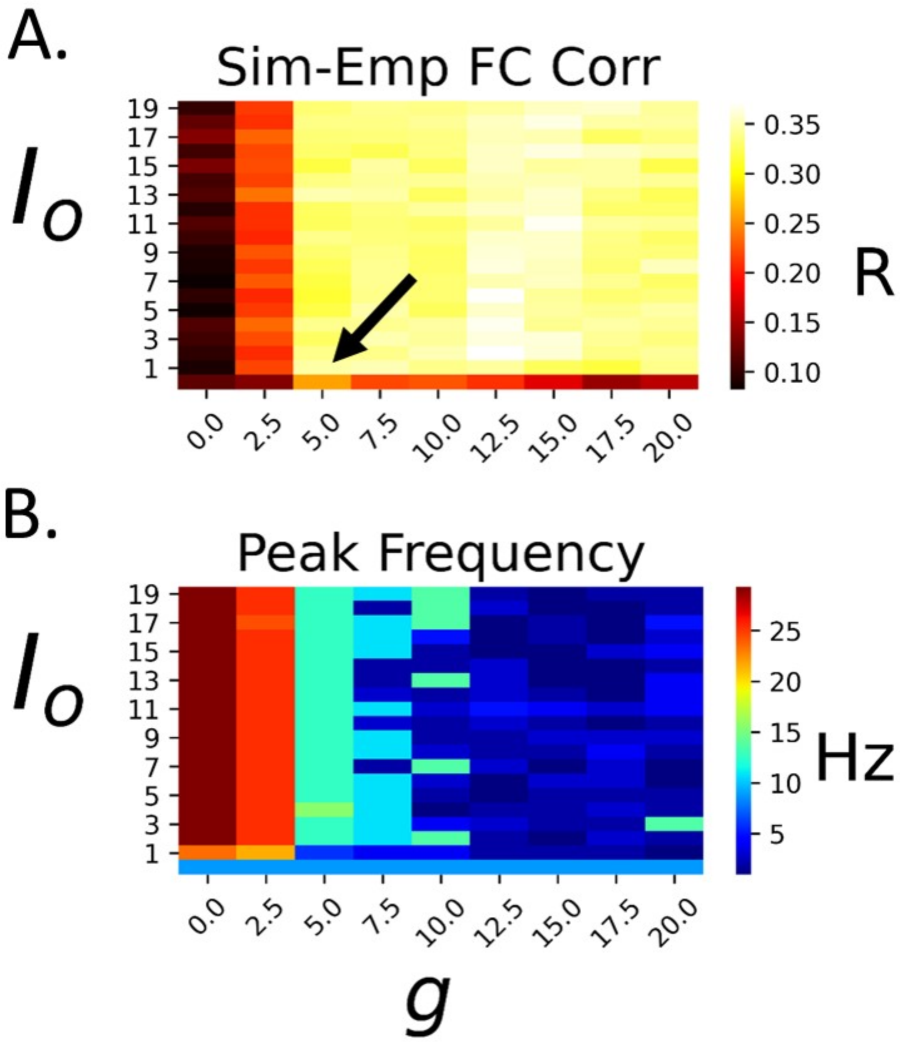

Figure S3. ***Parameter Space Exploration for*** g ***vs.*** I_o ***model parameters***. A) Frequency band-averaged simulated-empirical data functional connectivity (AEC) correlation (R). The black arrow indicates the onset of the high-fit zone. B) Peak region-averaged frequency at each parameter combination.

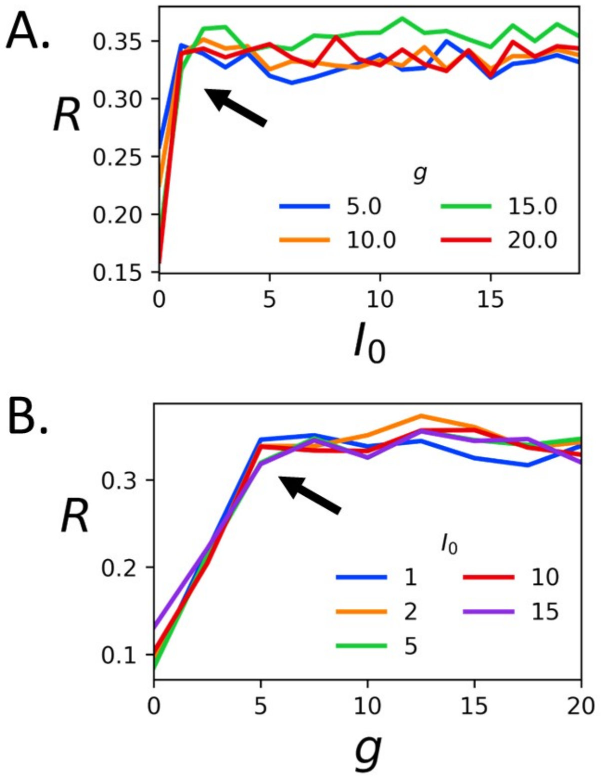

Figure S4. ***Parameter Space Exploration for*** g ***vs.*** I_o ***model parameters - selected values***. A) Band-averaged sim-emp correlation (R) for four values of g, across all 20 values of I_o. Each line corresponds to one of the *columns* of Figure S3A. B) R values for five values of I_o across all 20 values of g. Each line corresponds to one of the *rows* of Figure S3A. Black arrows indicate the 'shoulder', i.e. the I_o or g values at which R begins to asymptote.

The first clear feature in the PSE heatmaps in Figure S3a is a step-change in model fit values, occurring roughly at $g = 5.0$ and $I_o = 1.5$, indicated by the arrow. After that point, with increasing values of g and I_o, the band-averaged correlation with empirical functional connectivity asymptotes, visible as an undifferentiated block of high values in Figure S3A. This asymptotic behaviour is seen most clearly in Figure S4, which displays a few of columns (Figure S4a) and rows (Figure S4b) of Figure S3a as line plots. The 'shoulders' on these two plots are again indicated by arrows, showing that after a steep increase in model fit for increasing I_o and g, the fit levels remain consistent.

Accompanying these features in the fit values of Figure S3A are a number of corresponding paterns in the peak frequency heatmap in Figure S3b. First, at low g, the dominant frequency for $I_o = 0$ is alpha, which then transitions to gamma as I_o increases past 1. This mirrors the phase plane diagrams from Figure 2 of the main text. For $I_o > 1$ and higher values of g, however, the dominant frequency is gradually pulled down from 30+Hz to around 10Hz, and eventually at $g > 10$ to around 1-5Hz. Thus, it appears that the addition of long-range connectivity between cortical regions tends to 'pull down' the global average frequency into low-frequency regimes, and that this effect is stronger the stronger the global coupling. Note, however that the bifurcation point of $I_o > 1.0$ where the single-node model transitions from the alpha to the gamma frequency regime also represents the point at which the model reaches is optimal fit with empirical functional connectivity.

3 DEPENDENCE OF SIMULATION FEATURES ON RUN LENGTH

The simulations generating the MEG AEC functional connectivity plots in main text Figures 4 and 5 were run for 5 minutes of simulated time, so as to match the resting state MEG data against which we are comparing them. Other simulations, however were run for a shorter time (4 seconds for single-node power spectra in Figures 1,2,S1,S2; 20 seconds for whole-brain simulation PSEs in Figures S3, S4). These shorter run times were carefully selected to balance fidelity of the estimated quantities with computational load - particularly for the PSEs which required a large number of individual simulation runs. We found that single-node power spectrum estimates were always stable for 4 second runs, and did not deviate appreciate from those obtained from longer runs (data not shown). Likewise, for the whole-brain simulations, we found that features of AEC FC matrices matrices asymptoted by around 20 seconds, with negligible differences to those computed from longer run durations. This is demonstrated in Figure S4, which shows the same AEC matrices as displayed in Figure 5, for 4, 20, and 300 second simulation runs. As is visually clear from this figure, the AEC matrices at theta, alpha, beta, and gamma frequencies show negligible change for 20+ second runs (rows 2-4). The delta frequency matrices show globally stronger AEC at shorter runs, and are likely less well estimated for 4 second runs (which is unsurprising given that the delta oscillation period only allows for a handful of cycles in 4 seconds). The 'sim-emp FC Corr' metric used in the 20 second simulations from Figures S3 and S4 may therefore receive somewhat noisy contribution from the lowest frequency AEC. As this is only 1/5 of the total, however, with the other 4/5 (theta, alpha, beta, gamma) showing stable AEC estimation at 20s runs, we can reasonably expect these PSE results to closely approximate longer simulation runs.

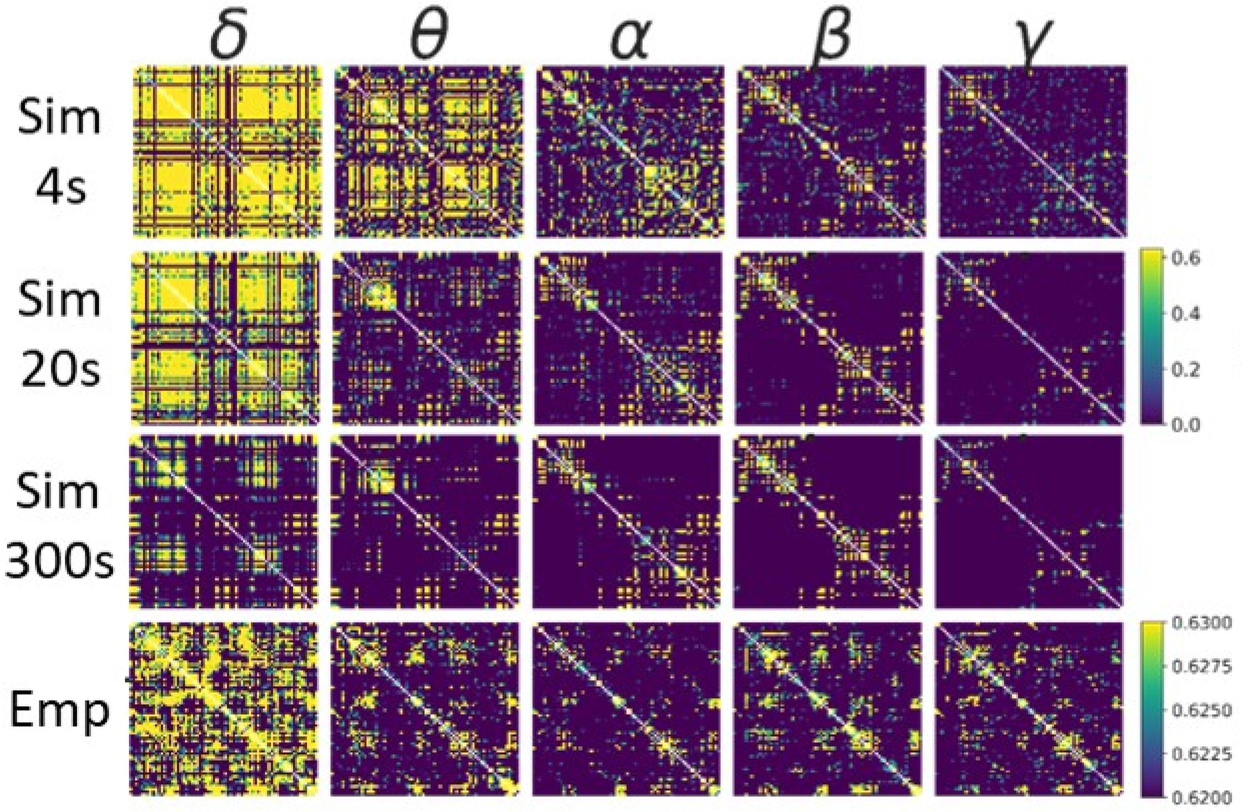

Figure S5. ***Influence of simulation duration***. Simulated data (as in Figures 4 and 5) for simulation durations of 4s (top row), 20s (second row), 300s (third row), along with empirical MEG data (fourth row). Columns indicate frequencies within which AECs are calculated. The simulation results are stablest for highest frequencies, and after 20s runs are highly similar to the outputs of (substantially longer) 300s runs.

4 FIELD SPREAD IN MEG INVERSE MODEL

To verify that the AEC patterns observed in our source-space MEG analyses were not attributable to signal spread within the electromagnetic source model, we repeated the AEC analyses on surrogate data. Because the forward and inverse models are specific to each subjects' brain anatomy and relation to the MEG sensors, this was done at the individual subject level, for one exemplary subject. The procedure was as follows: First, for all 8000 (4000 per hemisphere) dipole locations in the MEG source model, a pseudo-source time series was created by randomly sampling values from a uniform, random, unit-normal distribution. Next, these were passed respectively through the forward and inverse models computed from analyses of the original resting state data, yielding surrogate MEG sensor-level time series and re-projected MEG source time series. Finally, the source time series were averaged within each parcellation region, and AEC was calculated at each frequency band, followed by leakage correction, the same as with the source-estimated empirical MEG data. As with other analyses, these operations were performed using functions from the MNE software library (particularly, *mne.minimum_norm.apply_forward* and *mne.minimum_norm.apply_inverse*). Figure 6 shows AEC and leakage-corrected (AEC_{lc}) matrices following this process. As can be seen, The 'raw' random-noise-surrogate AEC matrices (top row of Figure 6) do show some spatial structure, as indicated in particular by the prominent near-diagonal and secondary diagonals. These spatial correlation signatures are not seen, however, in the leakage-corrected AEC matrices (bottom row of Figure 6). We therefore conclude that field spread does not contribute significantly to the MEG functional connectivity patterns.

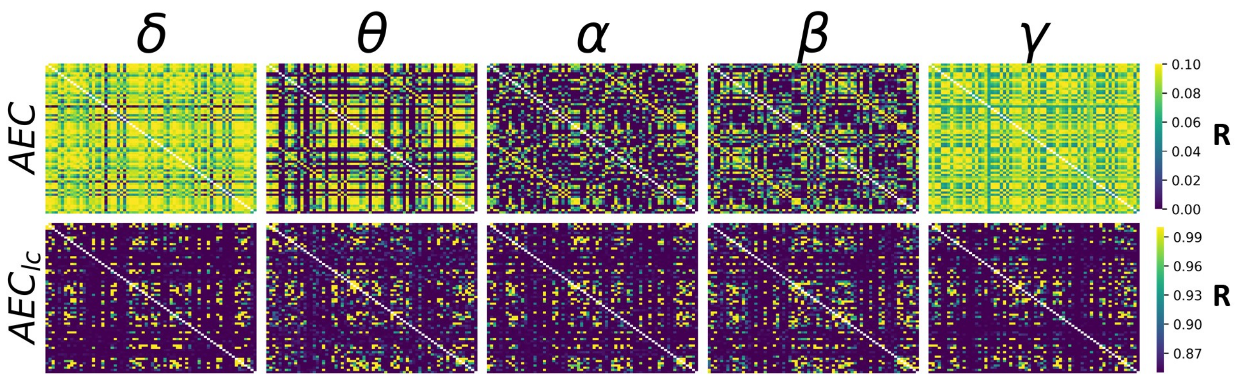

Figure S6. ***Influence of field Spread on source-space AEC FC.*** Original (upper row) and leakage-corrected (lower row) AEC functional connectivity matrices resulting from random noise surrogate source activity. All correlations in this figure are spurious by construction, since the input to the calculations originated from a random samples from a uniform distribution in source space. *Spatial* structure (such as strong secondary diagonals) in the correlation patterns is spuriously induced through the electromagnetic forward and inverse models. Fortunately, orthogonal leakage correction (lower row), as was used in our analyses of empirical MEG data, removes all spurious spatial structure from these randomly-generated AEC FC matrices.

.
